# Prototype of Instrumented Rock Bolt for Continuous Monitoring of Roof Fall Hazard in Deep Underground Mines

**DOI:** 10.3390/s23010154

**Published:** 2022-12-23

**Authors:** Krzysztof Fuławka, Witold Pytel, Marcin Szumny, Piotr Mertuszka, Bogumiła Pałac-Walko, Philipp Hartlieb, Michel Jakić, Michael Nöger

**Affiliations:** 1 KGHM Cuprum Ltd. Research & Development Centre, 2-8 Sikorskiego Street, 53-659 Wrocław, Poland; 2 Faculty of Geoengineering, Mining and Geology, Wrocław University of Science and Technology, 15 Na Grobli Street, 50-421 Wrocław, Poland; 3 Chair of Mining Engineering and Mineral Economics, Montanuniversitaet Leoben, Franz Josef-Strasse 18, 8700 Leoben, Austria

**Keywords:** roof fall hazard, ground control, stress monitoring system, axial stress, rock mass stability

## Abstract

Roof falls are currently one of the most dangerous threats associated with underground mining at great depth. Every occurrence of such an event poses a significant risk to the mining crew and disturbs the continuity of the mining process, which clearly affects the economy of the exploitation process. The development of a reliable monitoring system may significantly reduce the impact of eventual roof failure and will have a positive effect on the sustainability of the extraction process. Within this research study, a prototype of an instrumented rock bolt developed for continuous stress measurement is presented. The procedure of a 4-groove multilevel instrumented rock bolt is described and the calibration process is shown. Then, preliminary results of long-term in situ monitoring are presented. Based on the continuous monitoring of stress distribution within immediate roof strata, it was concluded that the developed instrumented rock bolt provides reliable results and is a very useful device, ensuring the possibility of early warning for miners about increasing roof fall risk.

## 1. Introduction

Ore exploitation performed in deep underground mines creates many threats, and roof fall is one of the most dangerous; therefore, properly selected support for mining excavation should be strictly adapted to local mining and geologic conditions [1,2,3,4]. The key factor affecting the type and required bearing capacity of roof support is the strength characteristics of rocks in the surrounding underground workings [5,6]. Other important factors are the depth of excavation [7,8], the presence of dynamic load in the surrounding area [9,10,11,12] and the utilized mining system [13,14]. All of these factors affect the stress level observed within the rock mass. With an increase in these stresses, eventual rock mass failure tends to be more destructive in terms of the amount of ejected rock and the scale of damaged workings [15,16]. Therefore, to prevent geomechanical risk intensification along with deteriorating geotechnical conditions, there is a necessity for the implementation of types of ground support that would be able to directly bear increased loads. 

Still, it must be highlighted that constructive housing selection for a particular condition may be performed in a reliable way only based on the data gathered with in situ measurements. Local characteristics of rock pressure, presence, level of shear stress and magnitude of axial forces are information which make it possible to find a compromise between costs, durability and the type of local support of underground workings.

For these purposes, numerous monitoring systems are utilised in underground mines [17,18,19,20,21,22,23,24,25,26]. Due to the harsh environment (humidity, dust), most monitoring systems are rather mechanical, such as convergence measuring systems and bed separation gauges [27]. The parameters measured with the use of convergence and bed separation indicators represent displacement which may not always be directly related to stress changes within the roof. Thus, electronic stress and strain systems with data acquisition seem to provide more accurate data about the real conditions of roof layers and the efficacy of used roof support. One of the most common devices used to determine the forces acting on the roof layers and roof support is the instrumented rock bolt [28,29,30,31,32,33]. This type of device allows the measurement of axial load and strains, high-strain zones and bending loads.

**Figure 1:**
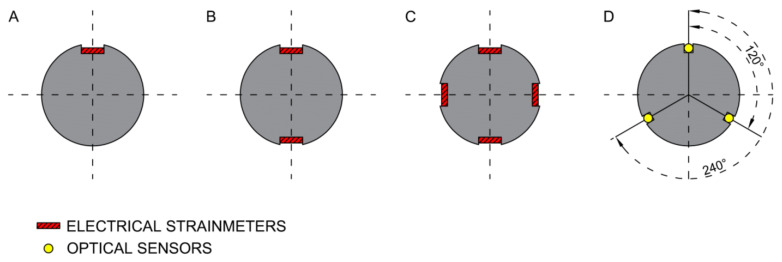
Development of strain sensor systems applied in measurements using instrumented rock bolts.

The history of technological development in the field of instrumented rock bolt construction dates back to the middle of the 1970s when Farmer [34] and Freeman [35] mounted an array of foil resistive strain gauges along the length of grouted rebar specimens (Figure 1A). This enabled them to assess the one-dimensional strain distribution along the rock bolts through the interpolation of the discrete measurements delivered by each strain gauge. In 1980, Farmer developed another type of instrumented appliance in the form of the hollow rock bolt, which contained four stainless-steel wires anchored at intervals of 0.38 m inwards from the base and measured deformations with a clip-on extensometer. Serbousek and Signer [36] developed the above solution by positioning similar strain gauges within a pair of diametrically opposed grooves made along the length of grouted rock bolts (Figure 1B). This allowed the strain distribution to be separated into axial and bending components through a comparison of the measured strain along opposing sides of the bolt. The same approach was utilized by Pytel [37], who used the finite difference method technique for bolt rod deflection (lateral) assessment using the elastic beam (rod) deflection second-order differential equation solution involving the values of bending moments and shearing forces based on in situ measurements. This was positively supplemented with numerical experiments performed on a large geometric scale and based on rock mass numerical models engaging the 3D finite element method, representing the roof bolting-pillar-floor systems in the in situ measurement site conditions. 

Similar consideration has been presented by Hyett et al. [38] who determined shear strain from the following equation:

(1) $$\varepsilon_{shear}=\frac{\left(\varepsilon_{A}-\varepsilon_{B}\right)}{2}$$

where: $\varepsilon_{A}$ and $\varepsilon_{B}$ are axial strain provided by strain gauges adhered in diametrically opposite grooves (A and B). Hyett et al. [38] also used Farmer’s [39] approach for bending strain assessment based on the central difference approximation, thus solving the second-order differential equation for displacement variation along the grouted bolt. 

Spearing et al. [40] tried a similar technique, replacing the short base-length resistive strain gauges with long base-length, from 200 mm to 500 mm, inductive strain gauges. Thus, owing that, the measurement results have been provided by “measurement zones” rather than “measurement points” as it was before. In 2019, the simpler plane two-groove system has been extended to the form of a four-groove system (Figure 1C), permitting assessment of the 3D stress–strain state within the bolts. This type of instrumented rock bolt was successfully used in Polish underground copper mines for assessment of shear and axial stress as well as for longitudinal and lateral displacement induced by blasting in the close neighbourhood [41]. 

In recent years, fibre optic systems have been rapidly developed and used to monitor the stability of mining infrastructure. Still, the use of fibre optic sensing had its beginning in the early twentieth century in the areas of tunnelling [42], civil engineering and structural health monitoring [43]. In addition, Schroeck et al. [44] discussed the use of fibre Bragg gratings (FBG) in rock bolt elongation monitoring in underground mines, but due to some technological limitations, their optical measurement system has not been more effective than systems utilizing electrical sensors. About seven years later, Naruse et al. [45] used distributed fibre optic sensors in rock mass movement monitoring in one of the Chilean underground mines. 

Perhaps the most intriguing aspect of optical sensing is the potential to use one optical fibre as both the lead and transducer for an array of measurements (i.e., a continuous or sub-continuous measurement sensor). From the success of Hyett et al. [46] and Forbes [47], it was decided to develop a new bolt-sensing solution with the capacity to capture the three-dimensional behaviour of rock bolts. The proposed solution considers the OFDR (Optical Frequency Domain Reflectometry) technology as the sensor but, unlike previous efforts, is arranged such that one optical sensor monitors three machined-out lengths along the bolt at 120 degrees from one another. Such a technical solution creates the capability for dividing strain into axial and lateral components, which in turn allows for lateral movement assessment. Moreover, shearing stress profiles along the length of a bolt support element may be simultaneously identified. A comparison of the existing ‘two-groove’ solution and the proposed ‘three-groove’ is visualized in Figure 1D [48]. However, FOS systems, with their advantages, also show specific features that call into question their wider use. One could mention here a limited range of strain measurement due to low strain limits of silica fibres as well as the insufficiently known direction of strain transfer from the surrounding rock, through the grout and to the fibre system [49]. In addressing such limitations, a validated earlier underground monitoring solution employing distributed resistive strain gauges has been used in this paper.

Within this paper, the process of development and testing of the autonomous stress monitoring system for continuous evaluation of geomechanical risk in the vicinity of active mining fronts in one of the Polish copper mines belonging to the KGHM was presented. The system is based on strain gauges and instrumented rock bolts, and it allows study of the three-dimensional process of deformation of roof layers, characteristics of shear and axial stresses, determination of warning levels based on measured data and bearing capacity of rock bolts utilized in a particular area. Data collected during long-term underground measurements were the base for the determination of the efficacy of local rock bolt support and the hazard level.

## 2. Materials and Methods

**Figure 2:**
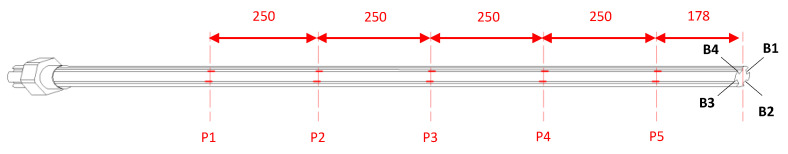
Scheme of the developed instrumented rock bolt.

The developed measurement system was based on the same 1.8 m long grouted rock bolts that are commonly used in Polish underground copper mines. To increase the quality and reliability of collected data, the measuring system consisted of 20 measuring points distributed along the entire length of the rock bolt and hidden in four grooves (5 in each groove) (Figure 2).

**Figure 3:**
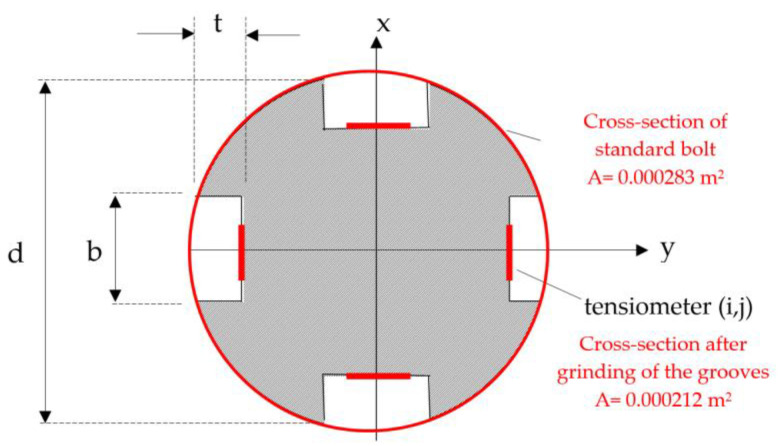
Geometry of the rock bolt and its accessories (t = 3 mm, b = 6 mm, d = 19 mm).

The relationship between the mechanical properties of an instrumented rock bolt and a standard bolt used in the analysed mining region should be considered from two points of view. Namely, the parameters of both rock bolts should be understood, as their load capacity will depend on their cross-sections. Generally, the reduction of the cross-sectional area of the rock bolt reduced the load capacity of the rock bolt, which is natural. It can be assumed that the decrease in load capacity was directly proportional to the change in the cross-section of the bolt. Knowing that the execution of the grooves reduced the cross-section of the rock bolt’s rebar by about 25% (Figure 3), it can be assumed that the load capacity of the instrumented rock bolt is about 25% lower compared to standard mining bolts used in the analysed mining region.

In turn, the deformation and strength parameters of the anchors remained the same or differed slightly when taking into account the process of groove preparation. Therefore, it can be assumed that the same deformation value was in the surrounding rock mass; using the basic relations of the theory of elasticity $\varepsilon =\frac{N_{i}}{EA_{i}}=\frac{\sigma }{E}$, it can be concluded that the dependence curve ε=f(σ) is identical if the rock bolt’s material is the same. On this basis, it can be expected that the measured deformation curves of the above-mentioned types of rock bolt in the same geological and mining conditions will not differ significantly, and thus it can also be concluded that the state of roof fall hazard signalled on the basis of observations is fully authoritative. It should be noted that a roof support consisting of standard rock bolts with a larger cross-section and higher load capacity may delay the failure of the roof; however, in this case, the axial stresses in the rod of the standard and instrumented rock bolt must simultaneously reach the value of the material yield strength. Nevertheless, it should be noted that each of the used instrumented rock bolts was calibrated before installation, and the relationship between the strains, axial forces and axial stresses were determined for the rock bolts with grooves. Knowing the stress-strain characteristics of the instrumented rock bolt, it was possible to determine the level of axial stresses in the rock bolt’s rod, indicating the state of roof fall hazard.

### 2.1. Construction of Instrumented Rock Bolt

**Figure 4:**
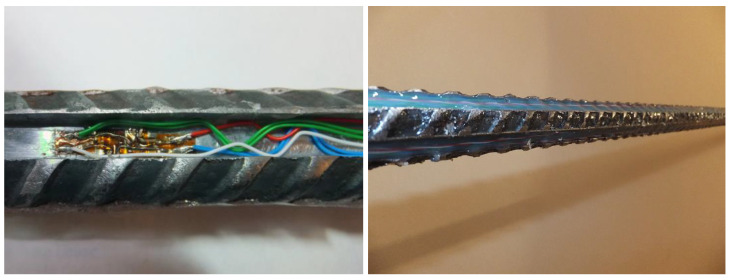
Connection of strain gauges on the anchor (**left**) and protection against weather conditions and impact (**right**).

The TF-1-2x/120 type foil strain gauges were chosen for the measurement system. These strain gauges are produced by the TENMEX Strain Gauge Laboratory from Łódź, Poland. These strain gauges, despite their complex structure, are one of the most technologically advanced strain gauges available on the Polish market. A single measuring point is made of a full strain gauge bridge consisting of four strain gauges with a resistance of 12 Ohms each. Before assembling the strain gauges, the surface was properly prepared. Connections of the strain gauges in Wheatstone bridges took place after their installation on the rock bolt. All elements of the measuring system (strain gauges, cables and solder pads) were located in the special grooves and protected against mechanical damage with silicone (Figure 4).

### 2.2. Data Collection and Signal Processing

**Figure 5:**
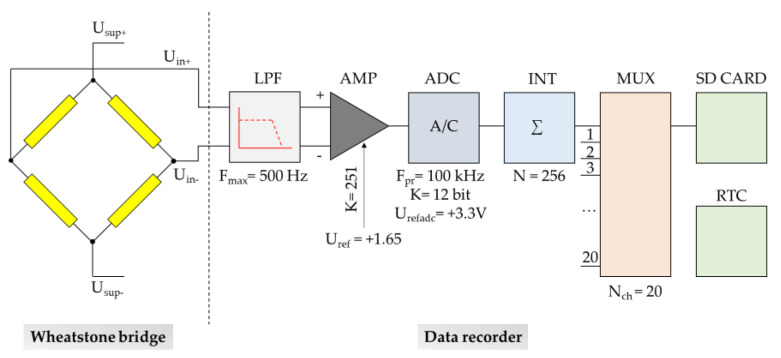
Block diagram of the recorder signal path.

The built-in recorder supplies all of the measuring points with power and converts the signals from the measuring points into a digital form that allows for the collection of data on the memory card. The signal diagram of the recorder for one measurement path is shown in Figure 5. On the recorder input, the signal is filtered with a low-pass filter, and then it is amplified and transformed from the differential signal U_in +_ and U_in-_ into a unipolar signal compatible with the input of the analogue-to-digital converter, i.e., 0 to +3.3 V:

(2) $$U_{out}=251\times \left(U_{in}\pm U_{in}-\right)+1.65$$

The analogue signal U_out_ is sampled in the converter with a frequency of 100 kHz. Then, the samples are integrated to improve the accuracy of the measurement. The data collection takes place after the signals from all measurement paths are multiplexed. Each entry on the card is labelled with the measurement time. Time measurement based on the real-time clock (RTC) is built into the microcontroller.

The signals from the Wheatstone bridges do not exceed the level of a few mV. Taking into account the fact that the dynamics of the analog-to-digital converter with a resolution of 12 bits are finite and amounts to approx. 72 dB, it is necessary to amplify the measurement signals with the use of the measuring amplifier to be able to register the smallest possible changes in stresses along the rock bolt’s bar. The gain of the measuring amplifier was set at 251 times. The maximum amplitude of the signal from the Wheatstone bridge at 1.2 V supply was ± 2.4 mV. After amplification and transformation into a unipolar form, we got signals in the range of:

(3) $$U_{min}=\frac{U_{refdac}}{2}-2.4\;mV\cdot 251=1.65-0.602\;V=1.048\;V$$

(4) $$U_{max}=\frac{U_{refdac}}{2}+2.4\;mV\cdot 251=1.65+0.602\;V=2.252\;V$$

It can be seen that the amplitude margin on the transducer is over 1 V for both polarities of the measurement signals. Leaving such margins is necessary due to the imbalance of the measurement system, called the offset voltage:

(5) $$U_{offs\;max}=\frac{U_{inmin}}{K}=\frac{1.048}{251}=4.2\;mV$$

Offset voltages are add to the measured signals and amplified equally. As long as the measured signal is within the amplitude window tolerated by the transducer, the offset voltage does not affect the quality of the actual measurement. The amplified measuring signal is sampled at a frequency of 1 kHz with a resolution of 12 bits. In order to improve the accuracy of the measurement, the samples are integrated with a fold of 256 times. This increases the dynamics of the measurement by

(6) 10·log256=24 d

Then, data from each of the 20 channels are collected and saved on the memory card with the real-time signature and the ID of the measurement channel.

### 2.3. Power Supply and Management

**Figure 6:**
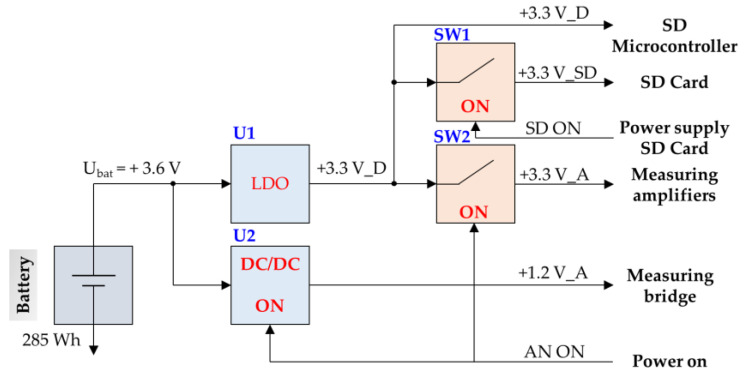
Power scheme of the recording system.

Taking into consideration the characteristics of the room-and-pillar mining system in KGHM mines, it was decided that the measuring system should be able to run only with use of a battery power supply. The length of a measurement session should reach up to several weeks. Therefore, minimization of the recorder’s power consumption plays a key role. Figure 6 shows the power supply diagram of the recorder modules.

The low power consumption of the monitoring system was achieved thanks to:

- the use of components with low energy consumption;
- the utilisation of an efficient DC/DC voltage converter for powering analogue circuits, including Wheatstone bridges;
- implementation of efficient power management.

The latter consists of switching on the power supply of the measuring circuits only when the system is triggered by the rapid increase of stress change amplitude. Additional energy savings can be obtained by turning on power to the SD card only during data saving. During the rest of the time, measurement data is buffered in the memory of the microcontroller until a sufficient amount of data is collected. Thanks to the use of the low-power integrated STM32 microcontroller platform and energy management strategies, the following energy consumption of the recorder was obtained depending on the operating mode. For the recording frequency of 10 Hz, it was 250 mW, while for 1 Hz, it was 110 mW. For comparison, continuously supplying 20 measuring bridges with a resistance of 120 ohms at 1.2 V would need 240 mW of power. When using a battery with a capacity of 285 Wh, the recorder working time without the need to replace the battery is 47 days in the 10 Hz mode and 108 days in the 1 Hz mode.

### 2.4. Data Calibration

**Figure 7:**
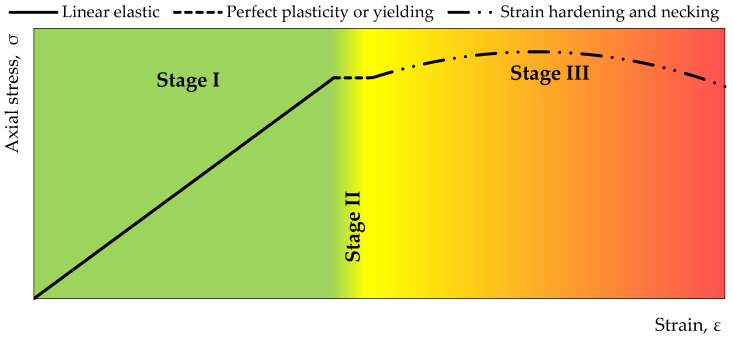
Stress-strain curve for steel used during the manufacturing of instrumented rock bolt.

The performed calibration of the installed measuring systems allows for converting the electrical data obtained directly from the forces acting on the rock bolt’s bar. Generally speaking, it can be stated that, ultimately, each strain gauge installed on the rock bolt can be assigned to a strictly defined value of force or stress. Laboratory tests have shown that the increase in the tensile load of 1 kN in the range of elastic loads corresponds to an increase of 176 units recorded with strain gauges with an accuracy of ~ +/− 5% (Stage I in Figure 7). Knowing the dimensions of the cross-section of the measuring anchor, the normal stress in the range of elastic strains “k” installed in the cross-section “*j*” can be calculated from the following equation:

(7) $$p_{\left(k,j\right)I}=\Delta \theta_{\left(k,j\right)}\cdot \frac{\xi }{A}=\Delta \theta_{\left(k,j\right)}\cdot \frac{0,0057}{1000\cdot A}$$

where ξ is the proportionality coefficient defining the relative axial load in the *j-th* cross-section’s increase of recorder indications on the strain gauge (*k,j*), which were obtained on the basis of calibration of measuring elements (strain gauges) during laboratory tests.

After exceeding the elastic limit, there is a temporary lack of stress increase and the material becomes plastic (Stage II in Figure 7). In this state, the stresses are determined by the relationship

(8) $$p_{\left(k,j\right)II}=4E-15\cdot \Delta \theta_{\left(k,j\right)}+408,68$$

Then, after exceeding the yield point, Stage 3 begins (Figure 7-red area). In this stage, the increase in strain becomes non-linear and is described by the relationship

(9) $$p_{\left(k,j\right)III}=-7E-07\cdot \Delta \theta_{\left(k,j\right)}{}^{2}+0,0356\cdot \Delta \theta_{\left(k,j\right)}+4,1761$$

### 2.5. Theoretical Basis

The developed instrumented rock bolt in its principal operation uses the well-known technique of resistance measurement with strain gauges, which is based on the relationship between changes in the resistance of a metal wire with the change in its shape. Strain gauge resistance sensors are usually used to measure deformations and, indirectly, stresses within the limits of the elasticity of the tested material. The value of the required unit strain ε is calculated based on the known value of the constant *k* for the strain gauge and the measured relative increase in electrical resistance $\frac{\Delta R}{R}\;$using the relationship

(10) $$\varepsilon =\frac{\Delta R}{R}/k$$

Knowing ε, based on Hooke’s law, it is possible to easily calculate the values of mainly compressive or tensile stresses along the rock bolt’s rod. In general, the principle of measuring deformation by means of strain gauges is to measure very small changes in electrical resistance caused by small deformations of the strain gauges glued to the element subjected to load.

In Polish copper mines, the room-and-pillar mining system with roof deflection is commonly utilised. As it was pointed out in Fuławka et al. [17], the dimensions of rooms and pillars are selected according to the local hardness of the rocks. The specific geologic structure, mainly within the roof stratum, favours the occurrence of geomechanical hazards of high intensity. The high strength and low deformability of the dolomite stratum leads to the accumulation of elastic energy within the main roof. The floor stratum, in turn, consists of much weaker sandstone. Under such conditions, pillars tend to be squeezed into the floor layers, which directly affects the forces observed in roof layers. Therefore, the main purpose of monitoring the behaviour of the rock next to active mining panels is to determine stresses in the horizontal and vertical directions of the roof stratum next to yielded pillars.

**Figure 8:**
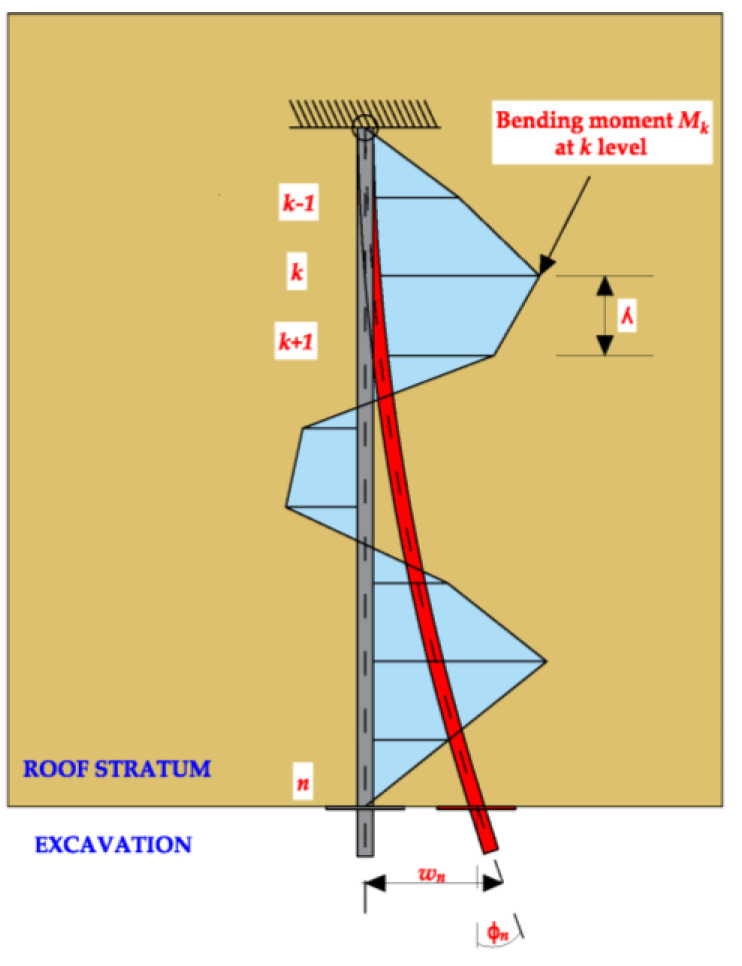
Rock bolt’s bar in a planned deformation state loaded with bending moments.

Knowing the force increment *ΔF* (or stress increment) in the rock bolts rod and the strain gauge indication increment *ΔR*, the equation describing the relationship *ΔF/ΔR* may be formulated. This type of relationship is the basis for determining the increments of axial forces and normal stresses, as well as bending moments along the rock bolt’s rod, as a function of time. Moreover, having the specified dimensions of the rod and the deformation parameters of the steel, the calculated loads along the rock bolt’s rod make it possible to determine deformations and displacements both in vertical and horizontal directions. In order to determine the deflection of the rock bolt’s rod (horizontal displacement) based on the calculated values of bending moments, one can use the discrete solution (finite difference method) of the differential equation of elastic beam deflection (Figure 8) in the following form:

(11) $$M_{x}\left(z\right)=-EI\frac{d^{2}w_{y}}{dz^{2}}$$

(12) $$M_{y}\left(z\right)=-EI\frac{d^{2}w_{x}}{dz^{2}}$$

where *EI* is the stiffness of the rock bolt’s rod, *M_y_(z)* and *M_x_(z)* are the bending moments acting around the mutually perpendicular axes y-y and x-x and passing through all strain gauges glued in a given plane and *w_x_(z)* and *w_y_(z)* are rod deflections directed parallel to the axes x-x and y-y.

A free supported beam or cantilever are the simplest static schemes utilized in the paper. Taking into account that the values of the rod’s bending moments are assessed based on continuous monitoring, using the differential equations for each k measurement level, two systems of the (*n*) algebraic equations may be formulated as follows:

(13) $$w_{x,k}=w_{x,\left(k-1\right)}+\lambda \phi_{y,n}+\frac{\lambda^{2}}{EI}\overset{n-1}{\underset{s=k}{\sum }}M_{y,s}\;k=1,\ldots ,\;n$$

(14) $$w_{y,k}=w_{y,\left(k-1\right)}+\lambda \phi_{x,n}+\frac{\lambda^{2}}{EI}\overset{n-1}{\underset{s=k}{\sum }}M_{x,s}\;k=1,\ldots ,\;n$$

where *w_x,k_* and *w_y,k_* are horizontal displacement in the x-x and y-y directions at the *k*-level, *λ* is the distance between measuring levels, *ϕ_x,n_* and *ϕ_y,n_* are the measured values of anchor rotation in the horizontal *n*-level and *EI* is stiffness of the rock bolt’s rebar. 

Shear forces directed parallel to the axes x-x and y-y, respectively, may be therefore described as follows:

(15) $$T_{x}=-EI\frac{d^{3}w_{x}}{dz^{3}}$$

(16) $$T_{y}=-EI\frac{d^{3}w_{y}}{dz^{3}}$$

Thus, the average shear stress within a rock bolt’s cross-section A, are: $\tau_{zx}≅\frac{T_{x}\left(z\right)}{A}$ and $\tau_{zy}≅\frac{T_{y}\left(z\right)}{A}$.

### 2.6. Description of the Site

**Figure 9:**
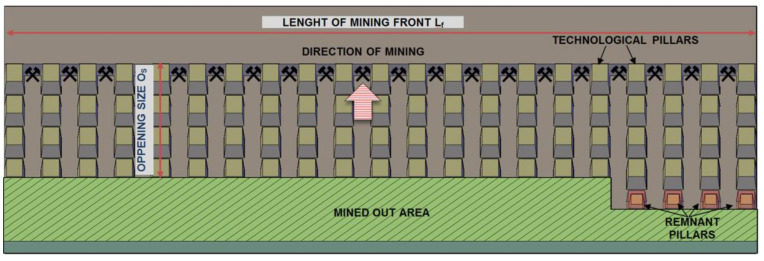
One-phase room-and-pillar mining system with roof deflection.

Preliminary measurements were performed in one of the mining panels in a Polish underground copper mine. Deposit excavation in this area is being performed with the use of the single-level room-and-pillar mining method with roof deflection. Generally, underground excavation with this system is based on cutting rock mass from rooms in such a way that the dimensions of their pillars allow them to yield. Therefore, the geometry of pillars is strictly dependent on the strength parameters of rocks. As a result, it is possible to gradually settle the roof, thus causing its deflection (Figure 9). 

**Figure 10:**
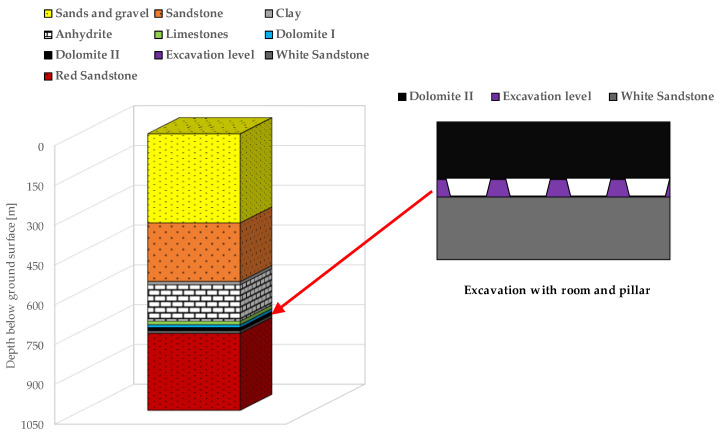
Geologic cross-section of the copper orebody.

The copper deposit in the research mining panel is classified as stratoidal and single-level. The ore bed includes grey sandstones and copper shales, while the deposit consists of shales with thicknesses in the range of 0.1 m to 0.6 m. Below shales, fine-grain quartzite sandstones with thicknesses up to 3.7 m are located. The average depth of the excavations is 750 m beneath the ground surface. Above the copper shales, there is a Zechstein carbonate series consisting of dolomites and limestones with a thickness of about 60 m. Over this stratum, dark grey and grey limestones are located. Above the carbonate rocks, there is a stratum of strong grey anhydrite with an average thickness of about 140 m. Higher, there are sandstones with a thickness of about 210 m. Finally, above the sandstones, there are sands and gravels with a thickness of about 340 m. In turn, the floor strata is formed of grey and red sandstones with a thickness of 10 m and 290 m (Figure 10).

The average compressive strength of rock within the roof stratum in the analysed mining panel was relatively high at *UCS* = 129.0 MPa. Still, roof strata in the area have a tendency to spall, which has in the past resulted in numerous roof falls. This hazard was particularly high within the mined-out zone.

## 3. Results

**Figure 11:**
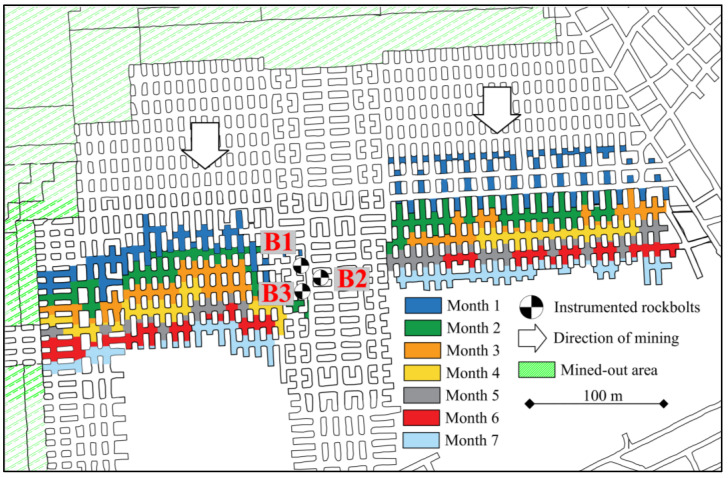
Map of the mining panel with stages of the operation’s progress.

Instrumented rock bolts were grouted in the forefront of the mining front to analyse the effect of the progressing excavation and dynamic load induced by blasting works on characteristics of roof stability (Figure 11).

### Results of Pilot Measurements

During the measurement, it was also assumed that the axial stress in each bolt’s rod and at each measuring level started at a value equal to zero. In order to analyse the roof fall hazard, axial stresses measured in four perpendicular directions were averaged according to the formula:

(17) $$\sigma_{avg\left(j\right)}=\frac{\sigma_{n\left(1,j\right)}+\sigma_{n\left(2,j\right)}+\sigma_{n\left(2,j\right)}+\sigma_{n\left(2,j\right)}}{4}$$

where $\sigma_{n\left(1,j\right)},\;\sigma_{n\left(2,j\right)},\sigma_{n\left(2,j\right)},\;\sigma_{n\left(2,j\right)}$ represent axial stresses measured at a rock bolt rod’s level no. *j* (MPa).

**Figure 12:**
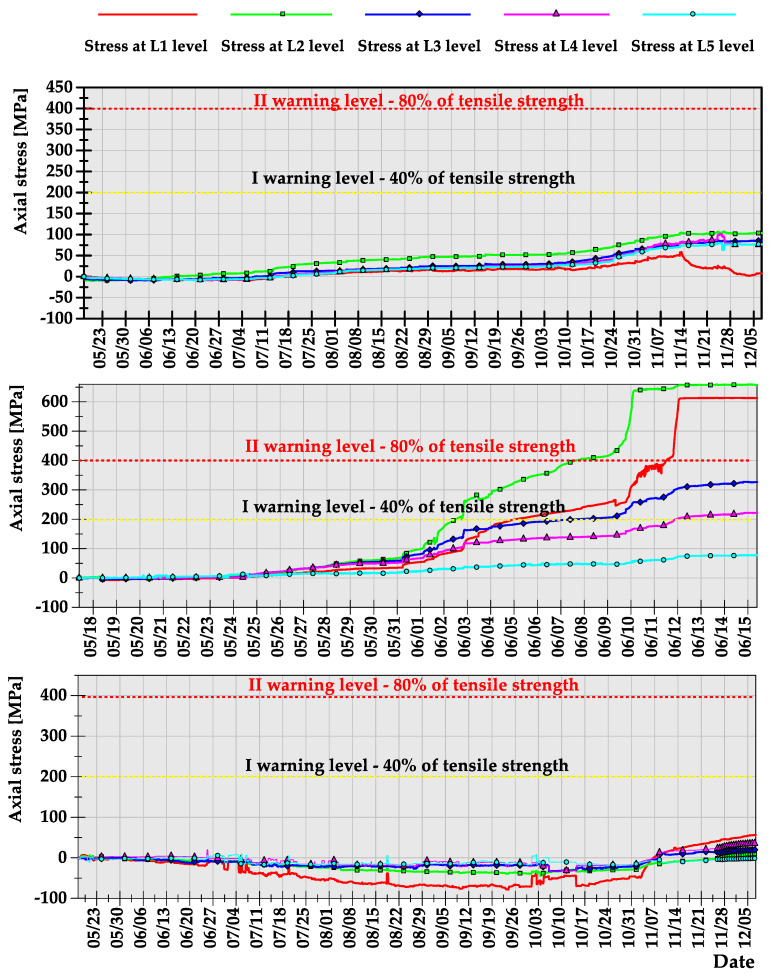
Axial stresses measured along rock bolts’ bars during the underground measurement.

During the preliminary measurements with three instrumented rock bolts, it turned out that roof fall hazard is very local in the areas located between technological pillars. The distance between subsequent rock bolts was about 30 m; however, despite that fact, the results of axial stresses recorded at subsequent levels were significantly different. The average axial stress distribution calculated based on the raw axial stress data has been presented in Figure 12. 

One may notice that rock bolts 1 and 3 were working continuously for 7 months of measurement, while within the area of rock bolt 2, roof fall occurred five weeks after the beginning of the measurement. What is important is the fact that, contrary to popular belief, the loss of stability of roof layers is not necessarily always a dynamic process. In fact, within the area of failure, based on the axial stress measurement, warning level I was indicated 2 weeks before loss of stability, while warning level II, which indicates the necessity of crew evacuation, was observed 1 week before the roof fall occurrence. In the case of rock bolts 1 and 3, the axial stresses after 7 months of measurements did not exceed the level of 150 MPa, which indicates safe geomechanical conditions in terms of the possibility of roof fall occurrence.

**Figure 13:**
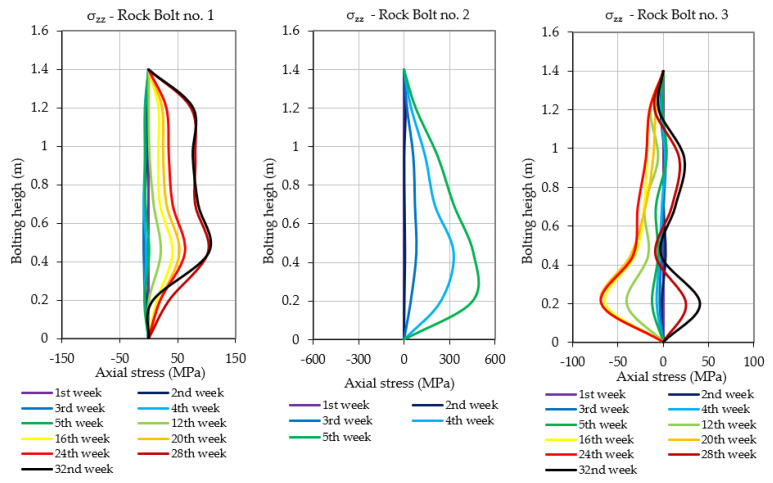
Axial stresses measured along rock bolts’ bars during the underground measurement.

**Figure 14:**
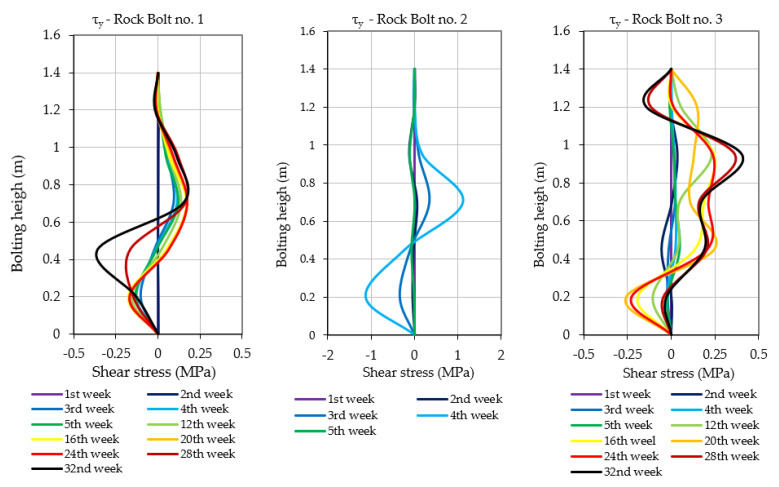
Shear stresses measured in rock bolts’ bars during the underground measurements.

**Figure 15:**
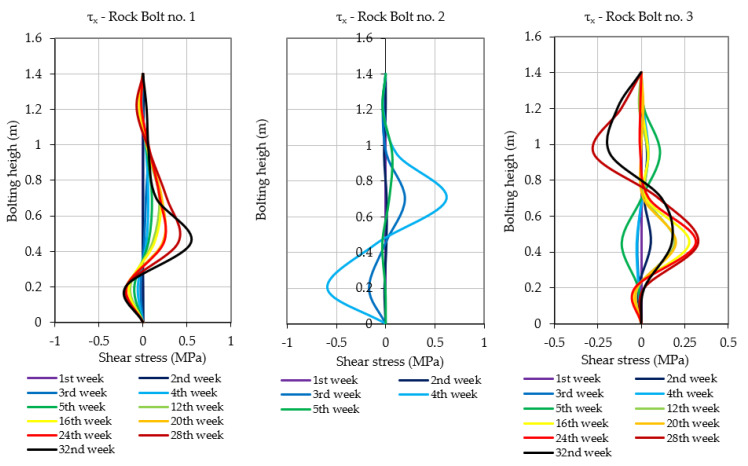
Shear stresses measured in rock bolts’ bars during the underground measurements.

As highlighted in Section 3, prototypes of instrumented rock bolts consisted of four perpendicularly placed grooves and five measuring levels. Multiple measurement levels allowed evaluating the scale of hazard by indicating the height at which the destruction/delamination of the roof layers occurs. Moreover, such a way of distributing measurement points within the rock bolt’s rod allowed not only for the determination of axial stresses, but also the calculation of shear stress acting on the rock bolt in the perpendicular directions x and y. The results of axial and shear stress acting in x and y directions, obtained after processing of raw signals, have been presented in Figure 13, Figure 14 and Figure 15. When analysing the distribution of axial stress, it may be observed that delamination of roof layers, such as in the case of instrumented rock bolt 2, occurred at the bolting height between 20 and 30 cm. In such cases, safety may be ensured by simply scaling off roof layers and by local reconstruction of the roof support. 

In the case of rock bolts 1 and 3, there is no necessity for implementation of roof support reconstruction or even implementation of additional roof support due to the low values of axial stresses at all measuring levels. In turn, based on the shear stress measurements, one may conclude that there was no risk of rock bolt failure in all three cases due to the very low level of horizontal stress within the analysed area. According to the analysis, the shear stress during the whole measurement did not exceed the level of 1.5 MPa in any of the measuring levels in the case of all three instrumented rock bolts. 

## 4. Discussion

According to preliminary results of long-term continuous measurements, it may be stated that developed prototypes of foil strain gauge-based instrumented rock bolts allowed reliable measurement of axial and shear stress in Polish underground copper mines, and it may be successfully implemented in regular use as a tool for continuous and autonomous geomechanical hazard evaluation. What is important is the fact that the proposed solution allows not only to indicate the hazard, but also to evaluate the scale of the threat by the indication of the level within the rock support where roof layers tend to delaminate, fracture and fail. On the other hand, 20 measuring points with the sampling rate of the recorder set to 1 Hz did not produce an amount of data that could not be effectively analysed, so it may be stated that a compromise was reached between the resolution and the amount of data. This is a visible advantage in comparison to fibre optic-based solutions where the large number of measuring points significantly affect the possibility of data transfer and management. Of course, there are also some disadvantages which have to be analysed in the future. The first disadvantage which must be mentioned is the relatively high cost of construction of such devices. In general, the price of foil strain gauges is still relatively high, and this affects the possibility of providing dense coverage for a mining area with monitoring devices. Another problem is the lack of interconnection between measuring devices and the lack of integration of data in one place, which results in a situation where data must be collected separately for each device. Therefore, it is worth analysing the possibility of providing cost-effective ways of data transfer to the main server where data from all devices in a particular region could be integrated and analysed. 

Of course, from data acquired in such a manner, many variables could be calculated and extracted, such as deformation change in time, strain along the bolt and utilization capacity. Furthermore, with more detailed analysis, there is a possibility to extract information about the state of the rock mass itself [50].

The results of the pilot measurements seem to be particularly significant from a risk management point of view due to the unambiguous indication of the possibility of predicting local stability loss even several days before the actual event by using instrumented anchors.

Nevertheless, further works aiming for integration between separate sensors are planned for the future. One way to combine all of the data acquired from intelligent bolts would require setting up a single Internet of Things (IoT) platform, which would allow for simultaneous analysis of data from different sensors. IoT platforms are often used to develop smart cities; they were designed to gather data from various sensors and combine them. Therefore, it would also make an excellent application for mining purposes [51]. Collected information could be examined and transformed into assertions about the level of safety in certain mining regions. What is important is the fact that an IoT platform allows rock bolts to be equipped not only with devices for monitoring of geomechanical parameters, but also for ventilation, vibration and many other parameters. The comparison of sensor data with threshold limit values that can be done for different gas levels would also include a study of the mine ventilation system and a method for predicting gas dispersion. To avoid conceptual blunders and to identify potential weaknesses, a more thorough investigation of such factors is necessary. This analysis should also include a review of their dependability and usability. Installation of several environmental sensors, however, can positively contribute to a safe working environment. Needless to say, collecting, sending and analysing this large amount of data imposes high requirements on the mine infrastructure not just in terms of connectivity and communication, but also in terms of engineering capacities and feeding back results and information to the workforce [50].

In terms of cost-effectiveness, there are a few monitoring rock bolt systems commercially accessible, but due to the high cost of production, none of them are affordable for large-scale operations in the mining industry; there is a high possibility of damaging sensors with blasting and other mining operations. Moreover, mining involves developing some areas for a certain amount of time until the ore is extracted, such as drifts and stopes that get blasted away; monitoring bolts would go to waste. In this case, implementing expensive measuring instruments is not a sustainable solution. That being said, there is still a big gap in the market for a low-cost, long-term monitoring solution in the mining industry. 

**Figure 16:**
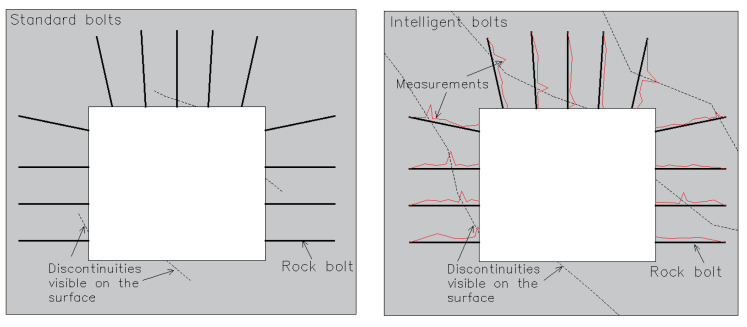
Possibility of identification of rock mass fracture with standard rock mass support (**left**) and instrumented rock bolts (**right**).

It is also worth considering the possibility of developing devices for simultaneous monitoring of many parameters. For chosen areas, geological data can be combined with bolt deformation data in order to predict rock mass behaviour and detect potential local instabilities. Useful input data for evaluating rock mass stability are joint orientation and positioning combined with the deformation values measured with rock bolts, which show possible weak spots in the rock mass (Figure 16). When enough data is available, machine learning techniques and big data analysis systems can be used to predict movements in the rock mass [52]. This calculation would also have to include the positioning of blasting areas and working machinery. It will be then possible to determine the effect of blasting events on the monitored rock mass and deformations over time by continuously monitoring the bolts’ deformations and knowing when and where blasting is taking place. This kind of data will be valuable for the entire mining process and for mine safety because predicting the position of instabilities can make the entire mining process safer and more predictable. In Figure 16, the advantage of spatial monitoring system is presented. On the left side, standard bolts are shown where discontinuities can only be visually inspected on the surface area, and on the right side are intelligent rock bolts that can monitor movements and show possible instabilities. Here, the red line shows the deformation of the bolt where discontinuities can be clearly seen by following the ‘’peaks’’.

## 5. Conclusions

Within this paper, the process of development and testing of strain gauge-based instrumented rock bolts has been presented. During the preliminary 7 months of long measurement, the usefulness of the proposed approach has been proved. According to preliminary data, the 1 Hz sampling rate was efficient enough to monitor the roof fall risk in the direct vicinity to the progressing mining front. In addition, the resolution of the used strain gauges allowed monitoring of even slight changes in stresses acting on the rock bolts’ rebar, which seems to be very useful, especially in the case of monitoring of stability within critical underground mining infrastructure, such as chambers with heavy machines, etc. What is most important is the fact that within the area of the roof fall, it was possible to indicate about 7 days before failure the necessity of crew evacuation. Still, based on long-term continuous measures, it was proved that the character of roof failure is very local, and there may be no visible symptoms of rock mass disintegration in the distance of about 20–30 m from the area of stability loss. Therefore, the place where instrumented rock bolts for axial stress measurement are planned to be installed has to be chosen wisely. In general, this kind of device should be used in places where the stability of the inner workings is of the highest importance, such as underground chambers, or in the regions within the mining panels in which ground control problems are expected. According to this study, the instrumented rock bolt’s prototype was robust and reliable. Still, implementation of large-scale monitoring systems to underground conditions requires that intelligent rock bolts must be low-cost and simple to install in order to become a viable option for the mining industry. 

In the next months, it is planned to implement solutions which will significantly reduce the costs of the instrumented rock bolts. It is also planned to connect different rock bolts into one monitoring system for continuous hazard evaluation. 

## Data Availability

The data presented in this study are available on request from the corresponding author.
